# Uncommon Muscle Complications in Diabetes: A Case Report on Diabetic Muscle Infarction

**DOI:** 10.7759/cureus.68874

**Published:** 2024-09-07

**Authors:** Twinkle Pawar, Sunny Malde, Sushrut Gupta, Vijay Jeyachandran, Pranjal Kashiv, Shubham Dubey, Kapil N Sejpal, Manish Balwani, Amit S Pasari, Charulata P Bawankule

**Affiliations:** 1 Medicine, Jawaharlal Nehru Medical College, Datta Meghe Institute of Higher Education & Research, Wardha, IND; 2 Nephrology, Jawaharlal Nehru Medical College, Datta Meghe Institute of Higher Education & Research, Wardha, IND; 3 Nephrology, Saraswati Kidney Care Center, Nagpur, IND

**Keywords:** acute muscle pain, diabetes mellitus complications, diabetic muscle infarction, dual antiplatelet, hemodialysis, mri diagnosis, muscle necrosis

## Abstract

Diabetic muscle infarction (DMI) is a rare but severe complication of diabetes mellitus (DM), characterised by acute muscle pain and swelling, primarily in the lower extremities. Prompt recognition and management are critical for preventing further complications. We report the case of a 54-year-old male with a long-standing history of type 2 DM and hypertension. The patient presented with acute, severe pain in the left calf, which had progressively worsened over several days. Physical examination revealed oedema and tenderness in the left calf. Laboratory investigations indicated muscle injury and inflammation, and MRI confirmed the diagnosis of DMI by showing areas of muscle inflammation. The patient was treated with hemodialysis, dual antiplatelet, and supportive care, including analgesics for pain management and stringent blood glucose control. He was advised to avoid excessive physical activity and was educated on the importance of medication adherence and lifestyle modifications. Regular follow-up was scheduled to monitor his progress and adjust the treatment plan as necessary. This case underscores the importance of considering DMI in patients with diabetes presenting with acute muscle pain. Early diagnosis and appropriate management are essential for preventing complications and improving patient outcomes. Clinicians should maintain a high index of suspicion for DMI to ensure timely and effective intervention.

## Introduction

Diabetes mellitus (DM) is a chronic metabolic disorder characterised by hyperglycemia resulting from defects in insulin secretion, insulin action, or both. It affects approximately 463 million people globally and is associated with numerous microvascular and macrovascular complications [1]. Diabetic muscle infarction (DMI) is a rare yet significant complication of DM, characterised by localised ischemic necrosis of skeletal muscle tissue, primarily affecting the lower extremities [2]. The exact pathophysiology of DMI remains incompletely understood. Still, it is thought to involve a combination of microvascular disease, thrombotic events within small vessels supplying skeletal muscle, and possibly underlying chronic inflammation and endothelial dysfunction [3]. DMI predominantly affects individuals with longstanding DM, often complicated by other microvascular complications such as diabetic retinopathy and nephropathy [4].

Clinically, DMI typically presents as acute, severe muscle pain and swelling, mimicking other conditions such as deep vein thrombosis or cellulitis. This challenging differential diagnosis underscores the importance of considering DMI in patients with DM who present with acute-onset, unexplained muscle pain, especially in the absence of overt trauma or infection [5]. Imaging studies, particularly magnetic resonance imaging (MRI), play a crucial role in diagnosing DMI by revealing characteristic findings such as muscle oedema and areas of necrosis [5]. Early diagnosis of DMI is essential as delayed recognition can lead to complications such as muscle contractures, chronic pain syndromes, and even limb amputation in severe cases [6]. Management of DMI primarily involves supportive care, including pain management with analgesics, optimising glycemic control to minimise further ischemic damage, and promoting physical rest to prevent exacerbation of muscle injury [7]. Long-term management focuses on comprehensive diabetes care to reduce the risk of recurrence and progression of microvascular complications, highlighting the importance of patient education, lifestyle modifications, and regular medical follow-up [8].

## Case presentation

A 54-year-old male presented with acute, severe pain in his left calf, which he described as progressively worsening over the past few days. The patient reported that the pain was more intense in the left calf compared to the right. His medical history was significant for type 2 DM and hypertension for many years. He had a history of diabetic retinopathy, for which he underwent laser photocoagulation in the past, and chronic kidney disease on maintenance hemodialysis, suspected to be secondary to diabetic nephropathy since 2020.

Upon physical examination, the patient was found to have a pulse rate of 80 beats per minute and a blood pressure of 130/80 mmHg. Notable findings included oedema in both feet, tenderness in the left calf, absence of pallor, facial puffiness, and icterus. The cardiovascular examination revealed normal heart sounds, while the respiratory examination showed clear lung fields. The abdominal examination was unremarkable; no renal angle tenderness or organomegaly was noted. The patient's laboratory investigations revealed (Table 1).

**Table 1:** Laboratory investigations of the patient CPK: Creatine phosphokinase; TIBC: Total iron-binding capacity; CRP: C-reactive protein

Laboratory Values	Observed Values	Reference Range
CPK total	194.3 U/L	25-200 U/L
Serum iron	43.19 µg/dl	60-165 µg/dl
TIBC	222.19 µg/dl	250-400 µg/dl
Transferrin saturation	19.44%	30-50%
Ferritin level	600.86 ng/ml	21.81-274.66 ng/ml
CRP	10.98 mg/L	0-6 mg/L

**Figure 1:**
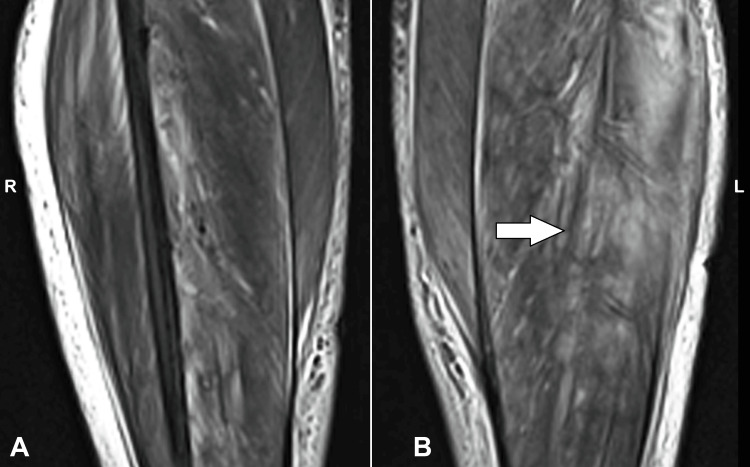
MRI axial section showing affected areas of the (A) right and (B) left muscles (white arrow) MRI: Magnetic resonance imaging

**Figure 2:**
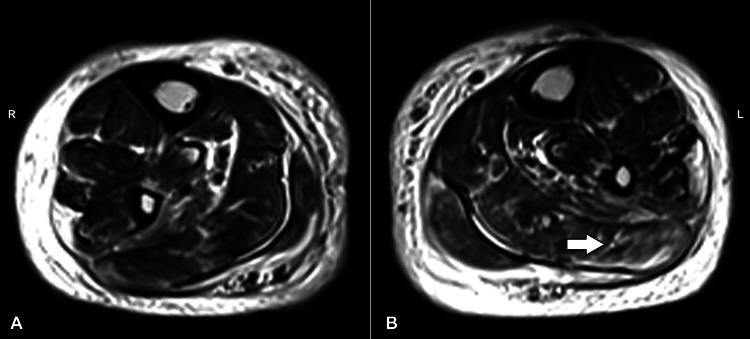
MRI coronal section showing affected areas of the (A) right and (B) left muscles (white arrow) MRI: Magnetic resonance imaging

The CRP level was elevated, suggesting an inflammatory response. Given the clinical presentation and laboratory findings, a diagnosis of DMI was suspected. An MRI of the bilateral calves was performed, which confirmed the diagnosis by showing areas of muscle inflammation (Figures 1-2).

The patient was advised to avoid excessive physical activity and was started on dual antiplatelets, hemodialysis, diuretics, antihypertensives, and supportive treatment, including analgesics for pain management and close monitoring of blood glucose levels to ensure optimal diabetes control. The patient was also educated about the importance of adhering to his medications and maintaining a low-sodium diet. Follow-up visits were scheduled to monitor the patient's progress and adjust the treatment plan as necessary. This case highlights the importance of considering DMI in diabetic patients who present with acute muscle pain, especially in the lower extremities. Early diagnosis and appropriate management are crucial in preventing complications and improving patient outcomes.

## Discussion

DMI is a rare but potentially debilitating complication of DM, characterised by ischemic necrosis of skeletal muscle tissue, predominantly affecting the lower extremities. The exact pathophysiology of DMI remains incompletely understood. However, it is believed to involve a combination of microvascular disease, thrombotic events, and impaired fibrinolysis within the small vessels supplying skeletal muscle [9,10]. The clinical presentation of DMI can often mimic other more common conditions, such as deep vein thrombosis, cellulitis, or diabetic neuropathy, leading to diagnostic challenges and delays [11]. In this case, the patient presented with acute, severe pain in the left calf, which progressively worsened over several days. This localised pain, often unilateral and exacerbated by movement, is characteristic of DMI and should prompt clinicians to consider this diagnosis in diabetic patients with similar symptoms [12].

Laboratory investigations play a crucial role in supporting the diagnosis of DMI. Elevated inflammatory markers, such as C-reactive protein (CRP), are often observed, as evidenced in the patient [13]. Imaging modalities, particularly MRI, are essential for confirming the diagnosis by revealing characteristic findings such as muscle oedema, inflammation, and areas of necrosis [14]. Management of DMI primarily involves supportive care aimed at pain management and optimising glycemic control to mitigate further ischemic damage [15]. Physical rest and avoiding activities that may worsen muscle ischemia are recommended to prevent complications and facilitate recovery [16]. Long-term management strategies for patients with DMI focus on comprehensive diabetes care, including strict glycemic control, management of associated comorbidities such as hypertension and diabetic nephropathy, and lifestyle modifications such as smoking cessation and regular exercise within tolerance levels [17]. Close monitoring and regular follow-up visits are essential to assess response to treatment, detect early recurrence, and adjust management strategies accordingly [18]. A similar case was reported of DMI in which leg pain was exacerbated during hemodialysis that may be secondary to diminished intravascular volume compartment due to ongoing ultrafiltration and diversion of blood from the intravascular compartment into the HD circuit. HD prescription was modified by increasing the duration, slowing the ultrafiltration rate, and reducing the blood flow rate [19].

## Conclusions

DMI, though rare, is a significant complication that can cause acute, severe muscle pain and swelling, predominantly in the lower extremities. This case report underscores the importance of maintaining a high index of suspicion for DMI in diabetic patients who present with unexplained muscle pain. Prompt diagnosis, primarily through clinical evaluation and imaging studies, and appropriate management are crucial to prevent further complications and improve patient outcomes. This case illustrates the need for awareness among clinicians regarding this uncommon yet serious condition, emphasising the role of comprehensive diabetes management in minimising the risk of such complications. Educating patients on adhering to treatment regimens, lifestyle modifications, and regular follow-up can aid in better management and prevention of DMI.
